# Bacteriophage cocktail application for *Campylobacter* mitigation - from in vitro to in vivo

**DOI:** 10.1186/s12866-023-02963-1

**Published:** 2023-08-05

**Authors:** Elisa Peh, Vanessa Szott, Benjamin Reichelt, Anika Friese, Uwe Rösler, Madeleine Plötz, Sophie Kittler

**Affiliations:** 1 Institute for Food Quality and Food Safety, University of Veterinary Medicine Hannover, Foundation, Hannover, Germany; 2 Institute of Food Safety and Food Hygiene, Freie Universität Berlin, Berlin, Germany; 3 Institute for Animal Hygiene and Environmental Health, Freie Universität Berlin, Berlin, Germany

**Keywords:** Phage therapy, Chicken, *Campylobacter*, Susceptibility, Antibacterial, Resistance, Drinking water

## Abstract

**Background:**

Effective strategies are urgently needed to control Campylobacteriosis, one of the most important foodborne gastrointestinal diseases worldwide. Administering bacteriophages (phages) is under evaluation as a possible intervention strategy in primary poultry production to reduce the public health risk of human infection. A major challenge is the translation of results from small-scale animal studies to large broiler flocks. In this study, the in vitro lytic activity of 18 *Campylobacter*-specific group II phages and 19 group III phages were examined singly, and in different combinations from the same group and from both groups using a planktonic killing assay. Based on these results, a combination of phage NCTC 12,673 (group III) and vB_CcM-LmqsCPL1/1 (group II) was selected for in vivo application in a seeder bird model to study its effectiveness under conditions as close as possible to field conditions. One hundred eighty Ross 308 broiler chickens were divided into a control and a treatment group. Ten days post hatch, seeder birds were orally inoculated with the *C. jejuni* target strain. Phages were administered via drinking water at a total concentration of 10^7^ PFU/mL four, three, and two days before necropsy.

**Results:**

Combining group II and group III phages resulted in significantly higher in vitro growth inhibition against the *C. jejuni* target strain BfR-CA-14,430 than single application or combinations of phages from the same group. The results of the animal trial showed that the application of the two phages significantly reduced *Campylobacter* counts in cloacal swabs. At necropsy, *Campylobacter* counts in colonic content of the treatment group were significantly reduced by 2 log_10_ units compared to the control group.

**Conclusions:**

We demonstrated that combining phages of groups II and III results in significantly increased lytic activities. The in vitro results were successfully translated into practical application in a study design close to field conditions, providing new data to apply phages in conventional broiler flocks in the future. Phage application reduced the fecal *Campylobacter* excretion and *Campylobacter* concentrations in the colon of broilers.

## Background

Campylobacteriosis has been the most frequently reported foodborne bacterial gastroenteritis in humans since 2005, with 127,840 notified cases in the European Union (EU) in 2021 [1]. It causes costs of circa €2.4 billion to the general public in the EU per year [2]. The poultry reservoir as a whole is considered to be the most important source of infection, being responsible for 50–80% of human cases [3]. *Campylobacter* (*C*.) *jejuni* and *C. coli* are the two most commonly detected species in reported human cases (88.4% *C. jejuni* and 10.1% *C. coli* in 2021) [1]. After *Campylobacter* introduction, the bacteria colonize the poultry ceca at concentrations up to 10^8^ CFU/g within days and enter the carcass by fecal contamination [4, 5]. In recent years, various strategies, such as the use of organic acids, plant extracts, or vaccines, have been investigated for their effectiveness in mitigating *Campylobacter* in poultry, with some successful treatments [6–9]. One mitigation strategy for bacterial infections that has received increasing attention in research, among policymakers and the general public is the use of bacteriophages (phages). Its development is largely driven by the urgent need for novel antimicrobials in the face of rising antibiotic resistance [10]. Phages are viruses that are the natural enemies of bacteria and are very specific in lysing bacterial host cells. Their specificity, in contrast to the relatively broad antimicrobial activity of antibiotics, offers the advantage that phage therapy does not disrupt the healthy gut microbiome [11–13]. However, phage therapy faces regulatory hurdles in the EU, as no phage product has yet received EU approval. Nevertheless, current developments in the EU indicate that phage therapy could be approved for veterinary medicine in the near future. Regulation (EU) 2019/6 on veterinary medicinal products, which came into force in January 2022, lists phages for the first time and defines them as “novel therapy veterinary medicinal products” [14]. The European Medicines Agency (EMA) recently issued a guideline describing regulatory, technical, and scientific requirements for the approval of phage products in veterinary medicine for public consultation [15].

Due to the phages’ characteristics, it is generally assumed that the application of phage cocktails has advantages over the use of single phages. The host range can be increased by combining phages that are effective against different isolates, allowing for broader application [16, 17]. Furthermore, there is evidence that combining phages with different lytic dynamic characteristics may impede the emergence of phage resistance in a bacterial population and thus ensure greater therapeutic success [18, 19]. *Campylobacter*-specific phages belonging to the most common family of *Myoviridae* can be divided into three groups, of which only group II and group III phages have been used in animal experiments to date. Phages from the two groups differ in their host spectrum and bind to different receptors on the bacterial surface [20]. In an in vivo study conducted by Hammerl et al. [21], it was observed that the successive application of phages from group II and group III reduced the fecal *Campylobacter* concentration in chickens by 3 log_10_ units, whereas the application of one or two phages from the same phage group resulted in a lower reduction (1 log_10_ unit) or no reduction. Despite the advantages of combining phages from different groups, most previous studies used single phages or phages from the same group to mitigate *Campylobacter* in chickens [22–28]. For successful translation of study results to practical application in the field, application techniques need to be easily integrated into the workflows of commercial broiler farms. However, previously published data on the use of *Campylobacter*-specific phages use application techniques not easily transferable to large broiler flocks, e.g., individual or successive application of phages to the animals [11, 21–23, 25, 27]. Therefore, studies are urgently needed that investigate the potential of combining different phages while considering possible hurdles of translation in their study design in order to successfully establish phage therapy in conventional broiler flocks in the future.

The aim of this study was to develop a phage cocktail with optimized efficacy in an in vitro selection scheme using a well-established planktonic killing assay (PKA) and to investigate its in vivo efficacy under conditions as close to field conditions as possible. For this purpose, a seeder bird model was used to imitate the natural spread of *Campylobacter* in conventional broiler flocks. The phage cocktail was administered to the broilers via drinking water, as this is the common route of drug application in large broiler flocks.

## Results

### Four phages were selected based on their group and planktonic killing assay results of single phages

The host ranges of phages were determined using an efficiency of plating (EOP) assay. The results show that the *C. jejuni* target strain BfR-CA-14,430 was susceptible to all 19 group III phages (100%) and to eight of the 18 group II phages (44%) included in this study (Table 1). The EOP values indicate how many plaques a phage forms on the target strain relative to the original host and are regarded as a measure of phage efficiency [29]. Of the group III phages, NCTC 12,673 and LmqsCPL1-2 showed the highest EOP values and formed 1.238 and 1.176 times more plaques on the target strain compared to their original host. Among the group II phages, the highest EOP values were observed for the phages LmqsCP218-2c2 (3.032) and LmqsCP288-2 (0.088).

A planktonic killing assay (PKA) was used to determine the ability of phages to inhibit bacterial growth in a liquid culture. Virulence indices were calculated from the data to facilitate comparability between phages and with EOP results. Of the group III phages, phages LmqsCP1-5 (0.46) and NCTC12673 (0.45) showed the highest virulence indices, while for the group II phages, the highest virulence indices were found for the phages LmqsCPL1/1 (0.45) and LmqsCP218-2c2 (0.44).

The two phages with the highest virulence indices were selected from each of the groups II and III phages for phage cocktail development and evaluation (LmqsCP1-5, NCTC 12,673, LmqsCPL1/1, LmqsCP218-2c2).

**Table 1:** Heatmap of efficiency of plating (EOP) values and virulence indices

Group III phages			Group II phages		
Phage	EOP^1^	Virulence index^2^	Phage	EOP	Virulence index
LmqsCP1-5	0.678	0.46	LmqsCPL1/1	0.004	0.45
NCTC 12,673	1.238	0.45	LmqsCP218-2c2	3.032	0.44
LmqsCP1-4	0.519	0.42	LmqsCP218-2c4	0.065	0.43
LmqsCP1-1	0.228	0.41	LmqsCP215-3	0.044	0.41
LmqsCP1-2	1.176	0.40	LmqsCP40-2	0.063	0.40
LmqsCP74-2c1	0.721	0.29	LmqsCPL1/2	0.003	0.38
LmqsCP264-3	0.203	0.28	LmqsCP288-2	0.088	0.27
LmqsCP81-1	0.237	0.27	LmqsCP288-3	0.026	0.26
LmqsCP235-1	0.095	0.27	LmqsCPL2	0.00	ND
LmqsCP134-3	0.403	0.27	LmqsCP209-1	0.00	ND
LmqsCP132-3	0.224	0.25	LmqsCP218-1	0.00	ND
LmqsCP244-3	0.098	0.24	LmqsCP218-2c3	0.00	ND
LmqsCP264-4	0.295	0.24	LmqsCP208-1	0.00	ND
LmqsCP225-3	0.438	0.24	LmqsCP253-2c1	0.00	ND
LmqsCP81-3	0.722	0.24	LmqsCP145-1	0.00	ND
LmqsCP73-1	0.035	0.23	LmqsCP49-1	0.00	ND
LmqsCP113-2	0.115	0.23	LmqsCP143-1	0.00	ND
LmqsCP136-2	0.101	0.23	LmqsCP215-2c1	0.00	ND
LmqsCP65-1	0.245	0.19			

ND, not determined

^1^ Efficiency of plating (EOP) values were determined by spot test

^2^ Virulence indices were determined using a planktonic killing assay in liquid culture

### Combinations of group III and group II phages were most effective in inhibiting ***Campylobacter*** population growth in vitro

**Fig. 1:**
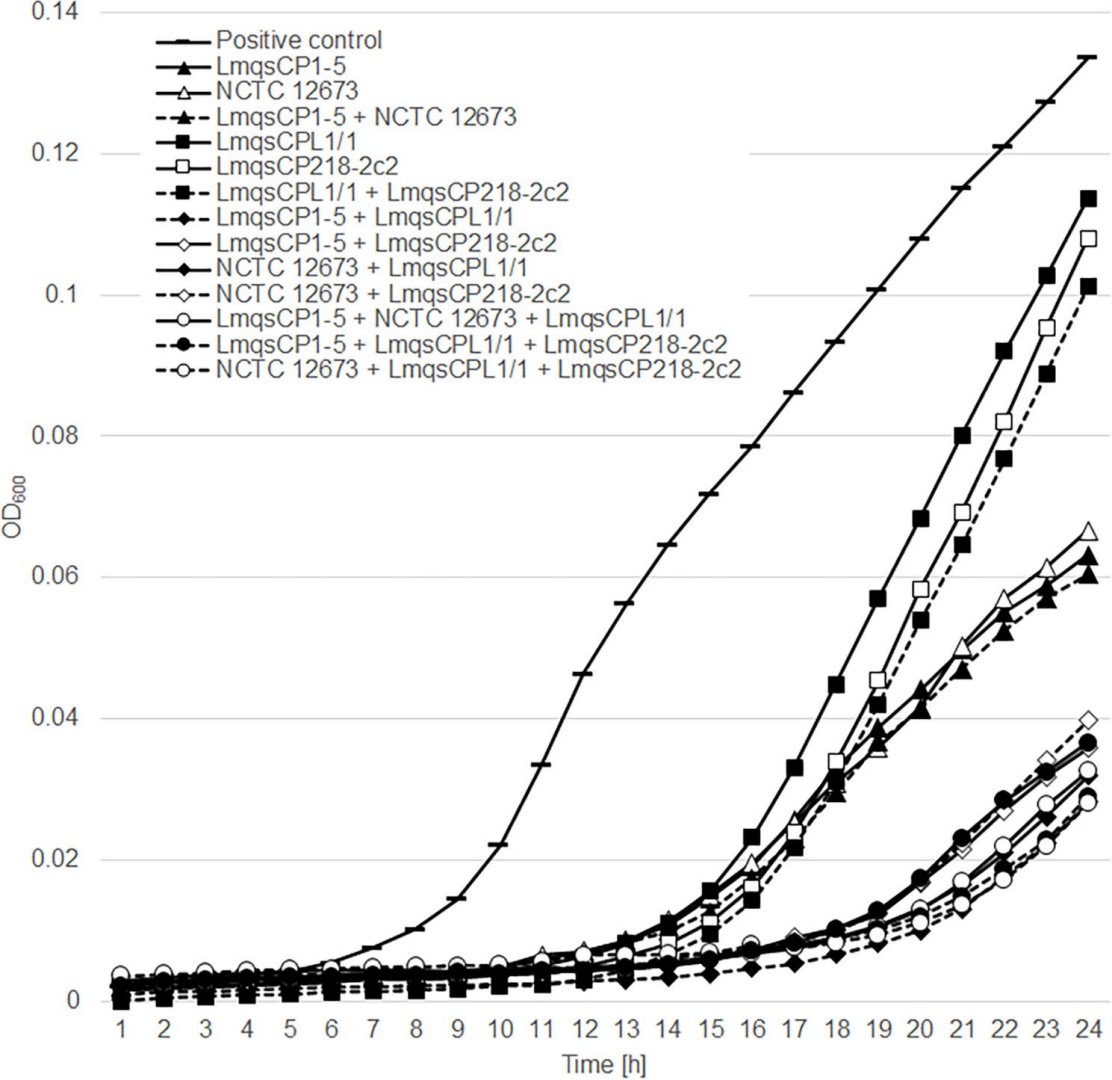
Growth curves of *C. jejuni* BfR-CA-14,430 in pure medium and in the presence of phages

Based on the results of the PKA of single phages, group III phages LmqsCP1-5 and NCTC 12,673 and group II phages LmqsCPL1/1 and LmqsCP218-2c2 were chosen for phage cocktail development. The four phages were mixed in all possible two to three phage combinations and their virulence indices were determined PKA. The results are shown in Fig. 1.

Phage cocktails consisting of phages from the same group (combination LmqsCP1-5 and NCTC 12,673; combination LmqsCPL1/1 and LmqsCP218-2c2) showed bacterial growth inhibition that was not significantly different from that of the respective individual phages (*p* > 0.05).

Combinations of group II and group III phages showed higher population growth inhibition, with the lowest OD_600_ values after 24 h of incubation (Fig. 1). The calculated virulence indices for phage cocktails consisting of group II and group III phages ranged between 0.83 and 0.89 and were significantly higher than virulence indices of single phages or combinations of phages from the same group (*p* < 0.001). A high virulence index indicates a high potential for bacterial growth inhibition of the tested phages. Phage cocktails consisting of three phages did not show higher virulence indices than cocktails comprising two phages.

Based on the high in vitro efficacy on the target strain and since the in vivo efficacy of phage NCTC 12,673 in reducing intestinal *Campylobacter* counts had been shown in previous studies [27, 28], a combination of group III phage NCTC 12,673 and group II phage LmqsCPL1/1 was selected for the animal trial (see below).

The group III phages LmqsCP1-5 and NCTC 12,673 and the group II phages LmqsCPL1/1 and LmqsCP218-2c2 were examined singly, and in different combinations from the same group and from both groups using a planktonic killing assay (PKA) at a multiplicity of infection (MOI) of 10. Growth was determined every hour by automatic OD_600_ measurements. The values represent the means of three measurements.

### In vivo results indicate a mitigating effect of the selected phage cocktail on ***Campylobacter*** counts in cloacal swabs and colonic content

Four days post hatch (dph), broiler chickens were confirmed to be *Campylobacter* free. Two days post oral inoculation (dpi) of the seeders (12 dph), all seeders of the treatment group and seven seeders of the control group were found to be *Campylobacter* positive by qualitative analysis of cloacal swabs.

**Fig. 2:**
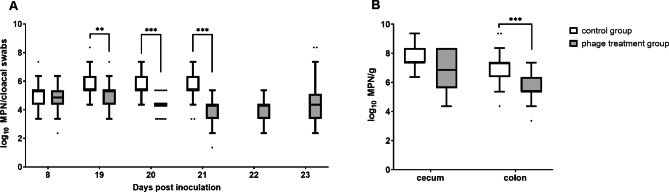
*Campylobacter* (*C*.) *jejuni* loads of 36 sentinels per group. *Campylobacter* concentrations were determined semi-quantitatively. Phages were continuously administered via drinking water 19, 20, and 21 days post inoculation (dpi) in the treatment group. (**A**) *C. jejuni* counts in log_10_ most probable number (MPN) in cloacal swabs from sentinel chickens. Cloacal swabs from 19 dpi were taken before phage application in the treatment group. At 22 and 23 dpi, the sentinels in the treatment group were exclusively sampled. (**B**) *C. jejuni* counts in log_10_ MPN per gram in cecal and colonic content of sentinels in the treatment and the control group after dissection (23 dpi). Medians (bold line) and significance levels (*p* values) between groups determined by the Mann-Whitney *U* test are indicated. * (*p* < 0.05), ** (*p* < 0.01), *** (*p* < 0.001)

Eight dpi of seeders (18 dph), semi-quantitative analysis of cloacal swabs revealed no significant differences between *Campylobacter* levels in the control and treatment groups (*p* > 0.05, Fig. 2).

The phage cocktail was administered 19 to 21 dpi to all animals in the treatment group. At 19 dpi, cloacal swabs were taken directly before the first phage application to evaluate the course of *Campylobacter* colonization prior to, during, and post phage application as accurately as possible. The cloacal swabs taken 19 dpi before the first phage application showed significantly (*p* < 0.01) lower *Campylobacter* colonization in the treatment group (*Median* (*Mdn*) = 5.362 log_10_ most probable number (MPN)/cloacal swabs, first quartile (Q_1_) 4.362 log_10_ MPN/cloacal swabs, Q_3_ 5.362) compared to the control group (5.362 log_10_ MPN/cloacal swabs, Q_1_ 5.362 log_10_ MPN/cloacal swabs, Q_3_ 6.362 log_10_ MPN/cloacal swabs). After administering the phage cocktail, the differences in the *Campylobacter* concentrations between the treatment and control group increased. At 20 and 21 dpi, a 1.0 log_10_ reduction (*p* < 0.0001) in *C. jejuni* counts was detected in the treatment group (*Mdn* = 4.362 and 4.362 log_10_ MPN/cloacal swabs) compared to the control group (*Mdn* = 5.362 and 5.362 log_10_ MPN/cloacal swabs). To statistically evaluate the course of *Campylobacter* colonization in the phage-treated experimental group, the Friedman test was applied. Results show that *Campylobacter* concentrations during and after phage treatment (21 and 22 dpi) were significantly lower than before phage treatment (19 dpi) (chi-sqaured test [4] = 19.03, p < 0.05, n = 36). In particular, *Campylobacter* concentrations differed significantly between 19 dpi and 21 dpi (z = 1.25, p = 0.008, r = 0.21) as well as between 19 and 22 dpi (z = 1.26, p = 0.007, r = 0.21).

After dissection, significantly lower *Campylobacter* counts by 2 log_10_ MPN/g were detected in colonic samples of the treatment group (*Mdn* = 5.362 log_10_ MPN/g) compared to the control group (*Mdn* = 7.362 log_10_ MPN) 23 dpi. However, *Campylobacter* counts in the cecal contents did not differ between the treatment group and the control group (*p* > 0.05).

### Re-isolates from the cecum showed the highest resistance rates

A total of 504 *Campylobacter* re-isolates were collected during the animal experiment from the phage-supplemented treatment group and tested for their susceptibility to the two applied phages. The two phages were administered continuously 19, 20, and 21 dpi. Re-isolates from 19 dpi were taken prior to administering the phage cocktail.

The proportion of re-isolates from cloacal swabs with reduced susceptibility to the group III phage NCTC 12,673 increased from 2.8% before phage application to 22.2% one day post phage application and decreased continuously thereafter to 12.5% at day 23 post infection (Table 2). Decreased susceptibility to the group II phage LmqsCPL1/1 was lower. From 19 to 23 dpi, all tested re-isolates were fully susceptible to the group II phage, except one 23 dpi isolate showing reduced susceptibility at day 23 post infection (1.4%).

The highest proportion of reduced susceptible re-isolates was found in cecal samples, with 23.6% (phage NCTC 12,673) and 2.8% (LmqsCPL1/1) of isolates showing reduced plaque counts in overlays and spot testing by 2.4 log_10_ and 2.7 log_10_, respectively. Re-isolates from colonic samples showed lower rates of reduced susceptibility of 15.3% to NCTC 12,673 and 0% to LmqsCPL1/1.

None of the re-isolates showed reduced susceptibility simultaneously to group II and group III phages.

**Table 2:** Proportion of *C. jejuni* BfR-CA-14,430 re-isolates from the treatment group showing reduced phage susceptibility (%)

phages	cloacal swabs					dissection	
	19 dpi	20 dpi	21 dpi	22 dpi	23 dpi	cecum (23 dpi)	colon (23 dpi)
NCTC 12,673^a^	2.8	22.2	18.1	16.7	12.5	23.6	15.3
LmqsCPL1/1^b^	0	0	0	0	1.4	2.8	0

Dpi, days post inoculation

^a^ Reduced plaque counts in overlays by a minimum of 2.4 log_10_ compared with the original *C. jejuni* target strain

^b^ Reduced plaque formation determined by spot testing by a minimum of 2.7 log_10_ compared with the original *C. jejuni* target strain

### Group II phage with high concentrations in cecum

**Fig. 3:**
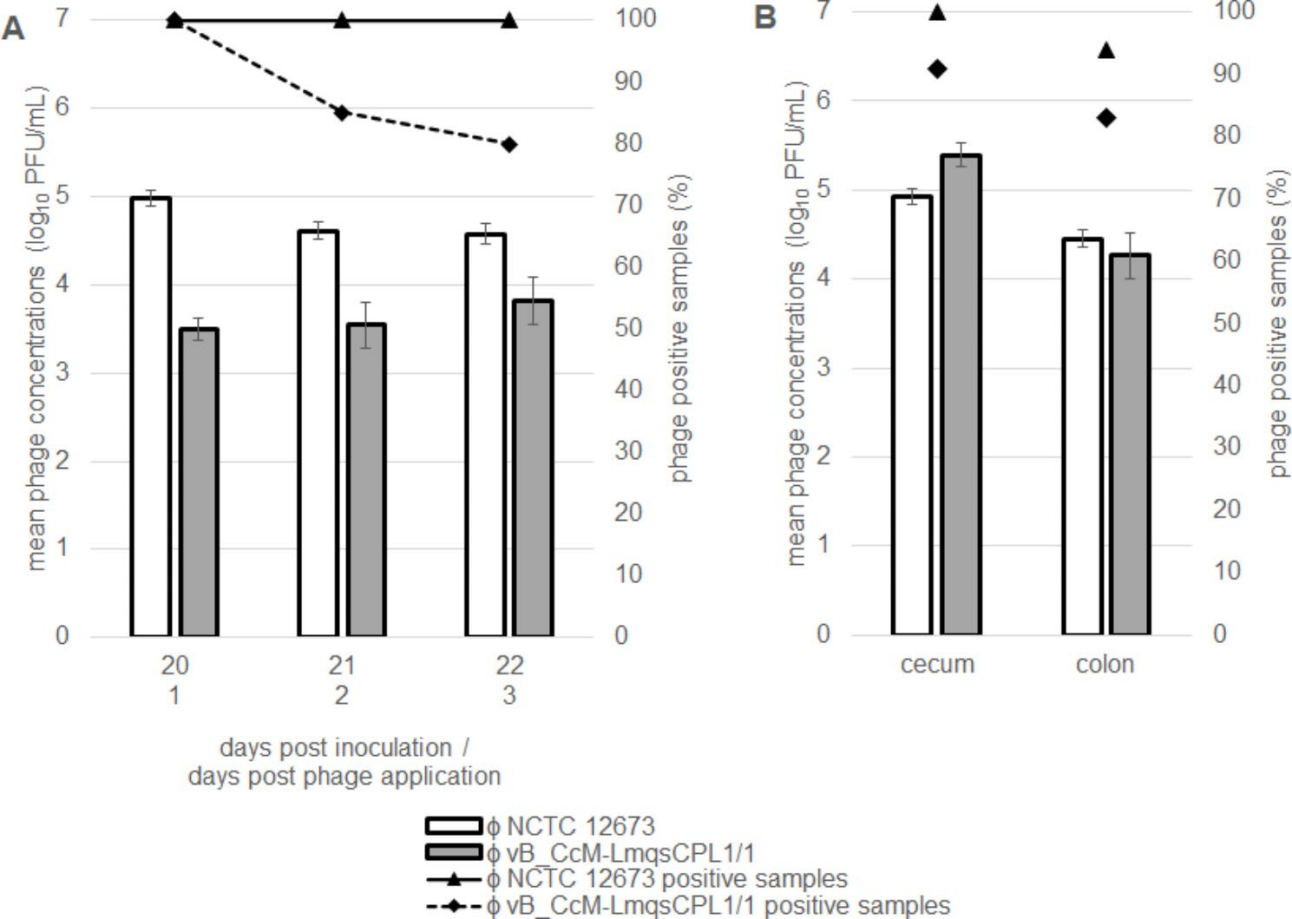
Phage concentrations (log_10_ PFU/mL) and percentage of positive samples (%) in the treatment phage group. Phage counts (**A**) in fecal samples 20, 21, and 22 days post inoculation (dpi) and (**B**) in intestinal content upon necropsy 23 dpi. Error bars show standard errors of the means

Phage concentrations were determined in the treatment group in fresh fecal samples collected 20, 21, and 22 dpi and after dissection in cecal and colonic samples of sentinels 23 dpi (Fig. 3).

The highest phage concentration was found for the group II phage in the cecum (5.4 log_10_ PFU/mL), exceeding the concentration of the group III phage by 0.5 log_10_ PFU/mL. In all other samples, the numbers of the group III phage were higher than those of the group II phage with concentrations ranging from 4.5 to 5.0 log_10_ PFU/mL. The percentage of group III phage positive samples ranged from 94% (colon) to 100%. Except for the cecal content, group II phage LmqsCPL1/1 concentrations were lower and ranged from 3.5 to 4.3 log_10_ PFU/mL. A total of 92% of the cecal and 83% of the colonic samples were phage group II positive.

### The group III phage showed a higher stability in drinking water than the group II phage

The phage cocktail was prepared in tap water for application via nipple drinker buckets and exchanged every 24 h. Water samples were taken immediately after preparing the phage-supplemented drinking water and 24 h later before exchanging the water to monitor phage stability during application.

The group III phage showed high stability with stable concentrations after 24 h, while the mean concentration of the group II phage was reduced by 2 log_10_ PFU/mL after 24 h in buckets with nipple drinkers.

## Discussion

Given the high impact of Campylobacteriosis on public health, effective mitigation methods are urgently needed. Mitigation strategies that target *Campylobacter* in primary production are considered to be very effective in reducing human Campylobacteriosis cases [30]. In this study, a phage cocktail was developed using a systematic in vitro approach and subsequently evaluated for its efficacy in vivo to reduce intestinal *Campylobacter* colonization in broilers.

For successful phage application in vivo, it is of utmost importance to select phages with a high efficacy against the target bacterium. There are different commonly used methods to investigate the efficacy of a phage for bacterial killing. In this study, we first determined the susceptibility of the bacterial target strain *C. jejuni* BfR-CA-14,430 to the panel of phages using an EOP assay. The target strain showed overall high susceptibility to the phages compared to *C. jejuni* isolates examined in previous studies [31, 32], with more phages from group III (19/19) than from group II (8/18) showing plaque formation. The EOP method compares the relative number of plaques that a phage forms on a target strain to the titer on a defined host strain and is a common method to quantify the efficacy of phages [29]. However, our EOP results differed significantly from those obtained using a liquid-based PKA, as the two methods ranked the phages in distinctly different orders according to their effectiveness. Similarly, Steffan et al. [32] did not observe a relationship between results of PKA and EOP in phage effectiveness against four *Campylobacter* strains. The differing results of the two methods could be explained by the two matrices, solid agar plates and liquid broth. The two matrices differ greatly in their spatial structure, which determines the degree of diffusion, mobility, and mixing, and might influence the outcome of phage application [33]. In contrast to our results, Haines et al. [34] observed a good correlation between results of the PKA and EOP used to determine phage efficacy against *E. coli* and *Klebsiella*. Nevertheless, both Haines et al. [34] and Steffan et al. [32] emphasized the advantages of PKA over EOP, as the PKA is described as less time and material consuming, and identified additional effective phages that did not form plaques in EOP. Moreover, unlike EOP, PKA can be used to study not only the efficacy of individual phages, but also that of phage combinations, which is essential for the optimization of phage therapy. In addition, the liquid medium of the PKA reflects much more the conditions in the intestinal contents of the animals: both represent a non-spatially structured environment, and the inhibitory effect of the phages is represented in a dynamic bacterial population [33]. For these reasons, results of the PKA method were used for developing phage cocktails.

Simultaneous application of the group II and group III phages inhibited the growth of the *C. jejuni* target strain significantly more than single phages or combinations of phages from the same group. Similar experiments were conducted in a previous study which investigated the inhibitory effect of group II and group III phages on the bacterial growth of a *C. jejuni* strain [21]. In contrast to our results, no differences were observed between the inhibitory effect of a group III phage alone and combined application of the group III phage with a group II phage when they were applied simultaneously. A significantly greater effect was observed only when phages of the two groups were applied successively; the reason for the observed differences in efficacy remains unclear [21]. A likely explanation for the greater growth inhibition by combined applications is the difference in receptor affinity, as group III phages bind to capsular polysaccharides of their hosts, while group II phages recognize their hosts by the flagellum [35, 36]. This might result in impeded bacterial resistance development, thus reducing bacterial population growth during phage application and leading to synergistic or additional bacterial lysis [37]. In general, a combined application of group II and III phages has the additional advantage of increasing the breadth of activity, since group III phages lyse *C. jejuni* exclusively, while group II phages lyse both *C. jejuni* and *C. coli* [32, 37, 38]. Due to its high efficiency in inhibiting the growth of the target bacterium and because the phage NCTC 12,673 has been successfully used in previous studies to reduce *Campylobacter* in vivo, the phage combination of LmqsCPL1/1 (group II) and NCTC 12,673 (group III) was selected for the animal trial [27, 28].

Increasing differences in *Campylobacter* load in cloacal swabs of the treatment and control group indicated a reducing effect of the phages. Due to a lower *Campylobacter* colonization in samples of the phage-treated experimental group, the in vivo efficacy could only be estimated by the increasing difference of bacterial concentrations in cloacal swabs between the treatment and control group. In previous studies, *Campylobacter* excretion via the cloaca and external contamination of feathers and skin has been shown to be an important source of cross contamination during both transport to the slaughterhouse and slaughter [39–44]. Thus, the reduced *Campylobacter* concentrations in feces observed in this study could result in reduced entry of the bacterium into the food chain. At necropsy, *Campylobacter* counts in colonic content of the treatment group were significantly reduced by 2 log_10_ MPN/g compared to the control group, while there were no significant differences in cecal concentrations. In contrast, Richards et al. [11] observed that the applied phage cocktail reduced *Campylobacter* counts more in the cecum than in the colon, resulting in 2.4 log_10_ units lower concentrations in the cecum compared to the control group two days after phage application. However, this reduction was not observed thereafter and five days after treatment, the difference between *Campylobacter* counts in the control and phage groups had decreased to 1.3 log_10_ units [11]. Limited reproducibility was observed in a field study conducted by Kittler et al. [28], as one trial demonstrated a reduction in cecal *Campylobacter* concentrations of 3.2 log_10_ units, while no significant reductions occurred in the other trials. However, decreases of 1 to 3 log units in the ceca of broilers were observed in other studies [11, 26, 27, 45]. The results indicate that there is still a great need for research to optimize the efficacy of phages and to achieve reproducible results for the control of *Campylobacter* in terms of public health targets. One possible strategy to enhance efficacy would be to combine phages with other methods, as multiple-hurdle approaches have been proposed to be more promising than single measures [46–48].

The reason for the lacking effect in the cecum remains unclear. Interestingly, the group II phage mean concentration was considerably higher in the cecum compared to colon and fecal samples and exceeded the phage III concentration exceptionally in this intestinal section. If the group II phage had exerted an antagonistic effect on the group III phage, the increased group II phage concentration in the cecum could have reduced the effectiveness of the phage cocktail. Similarly, a possible antagonistic effect of phage combinations was discussed in a study conducted by Hammerl et al. [21], as the application of a combination of two group III phages showed no reducing effect in vivo in contrast to a single phage that reduced *Campylobacter* counts. However, in another study, administering two group III phages in combination showed the same 1.5 log_10_ units *Campylobacter* reduction as a single phage [23]. Furthermore, in our study, antagonistic effects between the group III and group II phages are rather unlikely, based on the results of PKAs in vitro. Moreover, the previously mentioned study by Hammerl et al. (2014) demonstrated the efficacy of a mixed-group cocktail application in vivo.

High cecal *Campylobacter* concentrations after phage application can be explained based on our susceptibility testing results. The percentage of re-isolates showing reduced susceptibility was highest in the cecum with 23.6% (group III) and 2.8% (group II) compared to the colon and fecal samples. This could explain the reduced effectiveness of the phage cocktail in the cecum. Consistent with this, Fischer et al. [27] observed that after phage application, higher proportions of isolates with reduced phage susceptibility correlated with higher cecal *Campylobacter* colonization in broilers. The proportions of isolates showing reduced susceptibility described in the literature range from 4 to 43% for group III phages and from 2 to 13% for group II phages, which is consistent with our results [21, 22, 25–27]. Surprisingly, after an increase in reduced susceptibility to 22.2% of the re-isolates in cloacal swabs one day post phage application, the proportion of reduced susceptible isolates decreased continuously to 12.5% four days post phage application, even though selective pressure was maintained by applying the phage cocktail continuously over three days. In comparison, Scott et al. [24] observed that the sensitive isolate outcompeted the resistant strain only in the absence of phages. An explanation might be the fitness costs for *Campylobacter* isolates associated with reduced phage susceptibility as observed in previous studies [22, 24].

## Conclusion

In our study, we optimized the in vitro efficacy of phage cocktails in a targeted development process using a planktonic killing assay and demonstrated that combining phages of group II and III results in significantly increased lytic activities. The in vitro results were successfully translated into practical application in a study design close to field conditions, providing new data to apply phages in conventional broiler flocks in the future. Phage application significantly reduced the fecal *Campylobacter* excretion and *Campylobacter* concentrations in the colon of broilers. Further in vivo studies applying varying phage concentrations or combinations might be useful to increase the reduction potential, especially in the cecum.

## Materials and methods

### In vitro experiments

#### Bacterial strains

The whole genome-sequenced *C. jejuni* strain BfR-CA-14,430, kindly provided by the Federal Institute for Risk Assessment (BfR), served as a target strain for the in vitro and in vivo experiments.

For phage propagating and adjusting phage concentrations, *C. jejuni* strains NCTC 12,662 and NCTC 12,661, *C. coli* strain NCTC 12,667, and *C. coli* field isolate Cc084610 were used.

All *Campylobacter* strains were stored in cryotubes (Carl Roth GmbH & Co. KG, Karlsruhe, Germany, or Mast Diagnostica GmbH, Reinfeld, Germany) at -80 °C. For recovery, strains were streaked out on Columbia blood agar base supplemented with 5% sheep blood (Thermo Fischer Scientific Oxoid Deutschland GmbH, Wesel, Germany) and incubated at 42 ± 0.5 °C for 48 h under microaerobic conditions (5% O_2_, 10% CO_2_, 85% N_2_).

#### Phages

Phage NCTC 12,673 from the British phage typing scheme, 19 newly isolated group III phages, and 18 newly isolated group II phages previously published elsewhere [31, 32] were included in the present study. Phage suspensions were stored in SM buffer (5.8 g NaCl, 2.0 g MgSO_4_ × 7H_2_O, 50 mL 1 M Tris, adjusted to pH 7.5, filled up with distilled water to 1000 mL) at 4 °C.

#### Host range and efficiency of plating (EOP)

The susceptibility of the *C. jejuni* target strain BfR-CA-14,430 to the individual phages was determined using a conventional spot test in combination with an EOP assay as previously described [31, 32]. Briefly, liquid cultures of the *Campylobacter* strains were prepared in brain-heart infusion broth (Carl Roth GmbH & Co. KG) supplemented with 1 mM calcium chloride (CBHI) and incubated for two hours on a shaking platform (130 rpm) in an incubator at 42 ± 0.5 °C in a microaerobic atmosphere. Subsequently, 790 µL of the respective bacterial solution was transferred to 7.9 mL 0.7% NZCYM-soft agar (Carl Roth GmbH & Co., KG), thoroughly vortexed, and poured onto square plates containing 1.5% NZCYM agar. After solidification, 10 µL of a 10-fold dilution series of each phage was spotted on the overlay. The overlay plates were incubated for 20 ± 2 h at 42 ± 0.5 °C under microaerobic conditions and evaluated for visible plaques. All experiments were conducted in triplicate.

In order to determine the EOP, the ability to form plaques on the target strain was set in comparison to the respective phage host strains *C. jejuni* NCTC 12,662, NCTC 12,661, or *C. coli* Cc084610. Phages that produced plaques in two to three triplicates were used to calculate the efficiency of plating by dividing the concentration on the *C. jejuni* target strain by the concentration measured on the host strain of the respective phage.

#### Planktonic killing assay

Growth inhibition of the *C. jejuni* target strain BfR-CA-14,430 was investigated via optical density (OD) absorbance measurements at 600 nm (OD_600_) using a Tecan Spark automatic microplate reader with an integrated Gas Control Module (Tecan Austria GmbH, Grödig, Austria). Phage suspensions were adjusted to a concentration of 10^8^ plaque forming units (PFU)/mL and then diluted 1:10 in CBHI (10^7^ PFU/mL). Tests were performed as described by Steffan et al. [32] with minor modifications. Briefly, 200 µL of each phage suspension or 200 µL of CBHI (growth control) was added to the two wells of an F-shaped bottom 48-well microtiter plate (Sarstedt AG & Co. KG, Nümbrecht, Germany). For preparing the bacterial inoculum, the *C. jejuni* target strain was grown overnight and adjusted in 10 mol/L magnesium sulfate according to a McFarland standard of 5.0. Of this suspension, 1.5 mL were transferred to 25 mL of CBHI and incubated for four hours on a shaking platform (130 rpm). Subsequently, 3 mL of the liquid culture was adjusted to a McFarland standard of 0.5 using CBHI and diluted 1:100 in pre-heated CBHI (approx. 10^5^ colony-forming units (CFU)/mL). Two hundred microliters of this inoculum was transferred to each well of the microtiter plate containing 200 µL of the respective phage suspension (MOI of 10) or 200 µL of CBHI (growth control). The plate was stirred and incubated for 24 h at 42 ± 0.5 °C under microaerobic conditions. The OD_600_ was measured automatically every 60 min. The optical background of the CBHI was subtracted from OD_600_ measurements.

For comparison of bacterial growth patterns by OD_600_ measurement in the presence and absence of phages, virulence indices were calculated as previously described [32, 49]. The average values of three repetitions were used for calculations. First, the area under the curve (AUC) values were calculated for both the growth control and the phage treatments using the following equation:

i $$\text{AUC}\text{= }\sum _{\text{h}\text{=1}}^{\text{24}}\frac{\text{OD}\text{h+1}\text{ }\text{+}\text{ }\text{OD}\text{h}}{\text{2}}$$

Second, the virulence indices (*v*_*i*_) were calculated as follows:

ii $$\text{v}\text{i}\text{ }\text{= 1 - }\frac{\text{AUC}\text{phage}}{\text{AUC}\text{control}}$$

For development of phage combinations, the two phages observed to exhibit the highest virulence indices in single application were selected from group III (phage NCTC 12,673 and vB_CjM-LmqsCP1-1 (LmqsCP1-1)) and group II (phage vb_CcM-LmqsCPL1/1 (LmqsCPL1/1) and vb_CcM-Lmqs218-2c2 (Lmqs218-2c2)), respectively. All possible combinations comprising two (ratio 1:1) to three phages (ratio 1:1:1) were prepared using the four phages. The total phage concentration of the cocktails corresponded to 1 × 10^7^ PFU/mL to ensure comparability to the results of testing single phages. Planktonic killing assays of the combinations were conducted as described above. All experiments were performed in triplicate.

The combination of phage NCTC 12,673 and phage LmqsCPL1/1 was selected for the in vivo experiments.

### In vivo experiment

#### Housing

A total of 180 broiler hatching eggs of breed Ross 308 were obtained from a commercial hatchery and incubated for 21 days until hatch. Broilers were housed at a stocking density of 39 kg/m^2^. A commercial three-phase feed and tap water were supplied *ad libitum*. Water was provided in buckets with nipple drinkers, and, in the case of the treatment group, supplemented with the phage cocktail as described below. Facilities were equipped with an automated temperature control and a light regime to imitate conditions found in commercial broiler production.

#### Study design and sampling

Experimental procedures were conducted similar to those described by Szott et al. [50]. A total of 180 day-old broilers were divided into a control group and a treatment group receiving the phage cocktail (n = 90 broilers per group). To mimick the natural spread of *Campylobacter* from bird to bird in commercial broiler flocks, a seeder bird model was used. According to this model, individual broilers, called seeders (n = 18), were orally inoculated with the *Campylobacter* target strain. After intestinal colonization, seeders excreted *Campylobacter* via feces, naturally infecting the rest of the flock via the fecal-oral route as described by Awad et al. 2018 [51]. The naturally colonized broilers were divided into sentinels (n = 36), which were sampled at defined times for *Campylobacter* determination, and stocking density broilers (n = 36). The assignment of broilers to the three subgroups of seeders, sentinels and stocking density broilers was done randomly.

Four days post hatch (dph), cloacal swabs (Sarstedt AG & Co. KG) were taken from all 180 broilers and examined qualitatively for the presence of *Campylobacter* in accordance with DIN EN ISO 10272-1 as described below.

Ten dph, seeders of both groups were orally inoculated with 500 µL bacterial suspension containing approximately 10^4^ CFU of the *C. jejuni* target strain BfR-CA-14,430. Two days post inoculation (dpi), cloacal swabs were taken from seeders of both the control and treatment group and analyzed qualitatively in order to verify *Campylobacter* colonization.

Eight, 19, 20, and 21 dpi, sentinels of both groups were sampled by taking cloacal swabs. Sampling was performed in accordance with a standardized procedure. Briefly, the swabs were inserted into the cloaca, rotated five times, and removed. Cloacal swabs were analyzed semi-quantitatively in accordance with DIN EN ISO 10272-3 as described below. The cloacal swabs taken at 19 dpi were taken prior to phage application on the same day to determine initial *Campylobacter* concentrations before treatment. During the ongoing animal experiment, cloacal swab collection and processing were limited for organizational reasons 22 and 23 dpi and only the phage supplemented treatment group was sampled. In this way, the development of *Campylobacter* concentrations and phage susceptibility in the treatment group could be studied in more detail.

Dissection of sentinels was carried out 23 dpi (33 dph, average weight 2.0 kg). For sedation of the broilers, xylazine hydrochloride (1.75 mg/kg body weight, Xylavet 20 mg/mL, cp-pharma, Burgdorf, Germany), ketamine hydrochloride (43 mg/kg body weight, Ketamin 10%, Bremer Pharma GmbH, Warbug, Germany), and midazolam hydrochloride (0.85 mg/kg body weight, Midazolam 5 mg/mL, Braun, Melsungen, Germany) were injected into the pectoral muscle. After confirming deep anesthesia, the broilers were euthanized using ZKS poultry pliers (Corstechnology UG, Neerstedt, the Netherlands). Colonic and cecal contents were removed and diluted 1:8 in Preston broth. Subsequently, 10-fold dilution series were prepared and analyzed semi-quantitatively.

#### Composition and concentration of phages in drinking water

Due to the high virulence index determined in vitro, the combination of phage NCTC 12,673 and phage LmqsCPL1/1 was selected for application in vivo. Stock solutions of both phages were prepared with a concentration of 1 × 10^8^ PFU/mL and mixed at a ratio of 1:1. The phage cocktail was added to drinking water at a ratio of 1:10, reaching a final total phage concentration of 1 × 10^7^ PFU/mL. The phage cocktail was administered four, three, and two days before dissection (19, 20, 21 dpi). Every 24 h, the drinking water was changed and newly supplemented with the phage cocktail.

Assuming a daily water intake per broiler chicken of 200 mL, the total applied phage dose per broiler was approximately 6 * 10^9^ PFU over the three days of phage application.

#### Bacterial enumeration

Qualitative analysis of samples to investigate the presence of *Campylobacter* before inoculation and two days later was conducted in accordance with DIN EN ISO 10272-1 as described by Szott et al. [7]. Cloacal swabs were transferred into tubes containing 3 mL Preston Broth supplemented with Growth Supplement (Oxoid Deutschland GmbH), Preston *Campylobacter* selective Supplement (Oxoid Deutschland GmbH), and defibrinated horse blood (Oxoid Deutschland GmbH). After incubation at 37 ± 1 °C for 24 h in a microaerobic atmosphere, 10 µL of the suspensions were streaked out on modified *Campylobacter*-selective charcoal cefoperazone deoxycholate agar (mCCDA) plates prepared from *Campylobacter* blood-free selective agar base (Oxoid Deutschland GmbH) and CCDA selective supplement (Oxoid Deutschland GmbH) using inoculation loops (Sarstedt AG & Co. KG). Plates were incubated for 48 h and subsequently examined for typical *Campylobacter* colonies.

Semi-quantitative analysis of samples was carried out in accordance with DIN EN ISO 10272-3. Briefly, cloacal swabs were transferred to tubes containing 3 mL Preston broth and vortexed for three seconds. Subsequently, 10-fold dilutions in Preston Broth were prepared and incubated as described above. Dilutions were then streaked out on mCCDA plates, incubated for 48 h, and examined for bacterial growth. The highest dilution with confirmed *Campylobacter* growth was then used to calculate the MPN value using an MPN table modified in accordance with DIN EN ISO 10272-3:2010/Cor.1:2011(E).

Species confirmation was carried out using a Bruker Microflex system for matrix-assisted laser desorption ionization time-of-flight mass spectrometer (MALDI-TOF MS).

#### Phage susceptibility testing of ***Campylobacter*** re-isolates

For monitoring the phage susceptibility of *Campylobacter* after application of the phage cocktail, two colonies per sentinel of the treatment group were picked from mCCDA plates used for determining *Campylobacter* concentrations in cloacal swabs (19, 20, 21, 22, 23 dpi) and cecal and colonic content (dissection, 23 dpi). Colonies were transferred to tubes containing skimmed milk and stored at -80 °C as described earlier [28].

Susceptibility of *Campylobacter* re-isolates was tested separately for the two phages administered in the animal trial. The procedures were based on the methods described by Kittler et al. [28] and Fischer et al. [27] with minor modifications. Stock solutions of each phage were prepared in SM buffer, yielding concentrations of 2.5 × 10^3^ PFU/mL (NCTC 12,673) or 5.0 × 10^3^ PFU/mL (phage LmqsCPL1/1) using the *C. jejuni* target strain BfR-CA-14,430. Testing included the soft-agar overlay method as described above (phage NCTC 12,673) and a spot test (phage LmqsCPL1/1). For the latter, 10 µL of the phage suspension was spotted on overlays. After incubation for 24 h at 42 ± 0.5 °C under microaerobic conditions, plates were checked for plaque formation. Re-isolates with bacterial lawns without plaque formation were considered not susceptible to the respective phage.

#### Phage enumeration

To determine phage concentrations, 1 g each of 20 fresh fecal samples collected on the floor and 1 g of both cecal and colonic content obtained after necropsy from sentinels in the treatment group were transferred into tubes containing 9 mL SM Buffer and stored at 4 °C until further processing. For monitoring phage concentrations in drinking water, 50 mL were taken from the water of the treatment group immediately after adding the phage cocktail and 24 h later shortly before changing the water for each of the three days on which the phages were administered.

Concentrations of phages were determined using the soft-agar overlay method as described earlier [27]. Briefly, samples were centrifuged at 13,000 x g for 10 min, and the supernatant was filtrated through a 0.2 μm polyether-sulfone membrane (Carl Roth GmbH & Co., KG). Subsequently, 10-fold serial dilutions were prepared in SM buffer. Of these dilutions, 100 µL and 100 µL of an 18-hour *Campylobacter* culture adjusted to a McFarland Standard 3.0 were added to molten NZCYM overlay agar (0.7% agar agar) (Carl Roth GmbH & Co. KG), mixed thoroughly, and poured on plates filled with 20 mL NZCYM base agar (1.5% agar agar).

### Data analysis

Data derived from semi-quantitative analysis of *Campylobacter* counts during the animal trial were analyzed using SPSS software version 25.0 for Windows (SPSS, Inc., Chicago, IL, United States). The Shapirow-Wilk test was used to analyze data for normal distribution. Since the data were not normally distributed, the non-parametric Mann-Whitney *U* test was used for analyzing the data for significant differences. *Campylobacter* counts of the control group and the treatment group were logarithmized for analysis. *p*-values less than 0.05 were regarded as statistically significant. To ensure alpha error of 0.05, β-error of 0.18, and power of 0.80, a total of 90 animals per group were included in this study. In order to determine statistically significant differences, 36 animals were sampled during the experiment and the differences calculated by using a biologically relevant difference of delta = 1 log_10_ unit between *Campylobacter* counts of the groups, assuming a standard deviation of 1 log_10_ unit.

For the phage group, the course of *Campylobacter* concentrations in the cloacal swabs prior to, during and after phage application was additionally evaluated in SPSS using the Friedman test. The non-parametric Friedman test for dependent samples tests whether the central tendencies of several dependent samples differ. The Dunn-Bonferroni test was used as a post-hoc test.

Data of AUC values derived from PKA were analyzed using SAS 7.1. Analysis by the Shapiro-Wilk test showed that the data were normally distributed. The Bonferroni (Dunn´s) t-test was used to analyze the data for significant differences.

## Data Availability

All data generated or analyzed during this study are included in this published article.

## List of Abbreviations

AUC: Area under the curve
*C*.: *Campylobacter*
CBHI: Brain-heart infusion broth supplemented with 1 mM calcium chloride
CFU: Colony-forming units
dpi: Days post inoculation
EOP: Efficiency of plating
MALDI-TOF MS: Matrix-assisted laser desorption ionization time-of-flight mass spectrometer
*Mdn*: Median
MOI: Multiplicity of infection
MPN: Most probable number
OD: Optical density
PFU: Plaque forming units
PKA: Planktonic killing assay
rpm: Rounds per minute
